# MRI-Based Differentiation of Tumor Deposits and Lymph Node Metastases in Rectal Cancer: A Systematic Review of Diagnostic Performance

**DOI:** 10.3390/jcm15041390

**Published:** 2026-02-10

**Authors:** Paul-Andrei Stefan, Roxana-Adelina Stefan, Cosmin Caraiani, Lucian Marginean, Thea Diana Brad, Teodora Mocan, Codruta Gherman-Lencu, Dana-Monica Iancu

**Affiliations:** 1 Faculty of Nursing and Health Sciences, “Iuliu Haţieganu” University of Medicine and Pharmacy, Victor Babes Street number 8, 400347 Cluj-Napoca, Romania; stefan_paul@ymail.com (P.-A.S.);; 2 Radiology and Imaging Department, County Emergency Hospital, Clinicilor Street, Number 5, 400006 Cluj-Napoca, Romania; 3 Municipal Clinical Hospital of Cluj-Napoca, 3–5 Tabacarilor Street, 400139 Cluj-Napoca, Romania; 4 Department of Histology, “Iuliu Hațieganu” University of Medicine and Pharmacy, Victor Babes Street number 8, 400347 Cluj-Napoca, Romania; 5 Targu Mures, Department of Radiology and Medical Imaging, George Emil Palade University of Medicine, Pharmacy, Science, and Technology of Targu Mures, 38 Gheorghe Marinescu Street, 540139 Targu Mures, Romania; 6 Regional Institute of Gastroenterology and Hepatology “Octavian Fodor”, 19–21 Croitorilor Street, 400162 Cluj-Napoca, Romania

**Keywords:** rectal cancer, tumor deposits, lymph node metastases, literature review

## Abstract

**Background/Objectives**: Preoperative discrimination between tumor deposits (TDs) and lymph node metastases (LNMs) in rectal cancer on MRI is clinically critical but remains challenging. **Methods**: A systematic review following PRISMA guidelines was conducted, including studies using MRI to differentiate TDs from LNMs with histopathology as reference. Performance metrics such as AUC, sensitivity, and specificity were extracted. **Results**: Four retrospective studies (n = 344 patients) were included. Morphology-based MRI showed moderate diagnostic accuracy (AUC 0.72–0.76), whereas DCE-MRI and radiomics models demonstrated improved performance (AUC up to 0.86). Combined multiparametric approaches integrating DWI, DCE, and radiomics yielded the highest discriminative values. **Conclusions**: MRI-based multiparametric models improve discrimination between TDs and LNMs in rectal cancer. Integration of functional and morphologic features may enhance staging precision and guide therapy planning.

## 1. Introduction

Rectal cancer is one of the most prevalent gastrointestinal malignancies worldwide [1]. Tumor deposits (TDs) in rectal cancer are defined as carcinomatous nodules located in the mesorectal fat, within the lymphatic drainage territory of the primary tumor, lacking identifiable lymph node or vascular structures. While both TDs and lymph node metastases (LNMs) are important prognostic factors, they are pathologically distinct entities, and their prognostic impact is both independent and complementary [2,3,4,5].

Tumors exhibiting TDs are frequently associated with aggressive phenotypes, ultimately resulting in poorer oncologic outcomes and including disease-specific survival [6,7,8,9].

According to the American Joint Committee on Cancer (AJCC), tumor deposits are classified as N1c only in the absence of regional lymph node metastases. However, the TNM classifications lack specific guidelines for distinguishing TDs from LNMs [10]. Notably, when both TDs and LNMs are present, TDs do not alter the N category and are frequently omitted from formal nodal staging. This may result in underestimation of recurrence risk and disease burden [11]. Conversely, patients with TDs but without nodal involvement are less likely to receive adjuvant systemic therapy, despite evidence of unfavorable biology [12].

Reliable pre-treatment differentiation between TDs and LNMs is crucial for accurate prognostic assessment and to inform individualized therapeutic strategies. Therefore, they should be reported separately from nodal status [13]. However, distinguishing TDs from LNMs on pre-treatment MRI remains challenging, as no definitive imaging criteria have been universally validated for this purpose [14,15]. Radiologically, rounded mesorectal nodules are typically interpreted as LNMs, whereas irregular, heterogeneous nodules, especially those intersecting vascular or lymphatic structures, may be suggestive of TDs. However, the interpretation of medical images is subjective, as Rutegard et al. reported that approximately 44% of peritumoral nodules initially suspected to represent LNMs on MRI were later classified histologically to be TDs or nodular extramural venous invasion (EMVI). Histopathology remains the reference standard for this distinction, but its utility is retrospective and cannot inform preoperative management decisions [16]. As a result, the presence of TDs is often underrecognized in radiologic staging, potentially leading to an underestimation of disease burden and patient risk [17,18,19]. Advanced MRI sequences, particularly diffusion-weighted imaging (DWI) and dynamic contrast-enhanced MRI (DCE), offer functional insights into tissue cellularity and perfusion that may better reflect the underlying biological differences between the two entities, by providing surrogate markers for tissue cellularity and perfusion, respectively [20]. Furthermore, radiomic and texture-based analyses represent promising adjuncts in this context. These approaches extract high-dimensional quantitative features from imaging data. However, few studies to date have explicitly developed radiomic models for this specific diagnostic task [16,21].

Given the prognostic relevance of TDs and the ongoing imaging challenges they present, a consolidated synthesis of the available evidence is warranted. We therefore conducted a systematic review of published studies evaluating MRI-based methods for preoperative differentiation of TDs from LNMs in rectal cancer.

## 2. Materials and Methods

### 2.1. Literature Search and Study Selection

**Figure 1:**
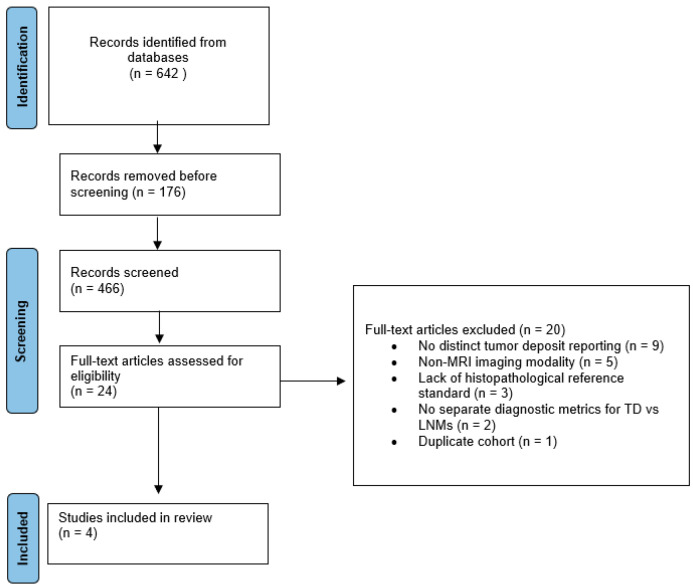
PRISMA 2020 flow diagram illustrating the study selection process.

This systematic review was conducted in accordance with the Preferred Reporting Items for Systematic Reviews and Meta-Analyses (PRISMA) 2020 guidelines, and the study selection process is summarized in the PRISMA flow diagram (Figure 1). A comprehensive literature search was performed across four major databases (PubMed, Embase, Scopus, and Web of Science) to identify studies published between January 2005 and November 2025 evaluating MRI-based preoperative differentiation of mesorectal tumor deposits from lymph node metastases in rectal cancer. The search strategy combined controlled vocabulary terms (MeSH and Emtree, where applicable) with free-text keywords related to rectal cancer, tumor deposits, lymph node metastases, and magnetic resonance imaging. A representative PubMed search string was: (“rectal cancer” OR “rectal adenocarcinoma”) AND (“tumor deposit” OR “mesorectal deposit*”) AND (“magnetic resonance imaging” OR MRI). Equivalent adaptations of this strategy were applied to Embase, Scopus, and Web of Science. In addition, reference lists of included full-text articles were manually screened to identify further eligible studies.

Titles and abstracts identified through the database search were independently screened by two reviewers (CGL and TDB) to assess eligibility. Full-text articles of potentially relevant studies were subsequently reviewed independently by the same reviewers. Any disagreements regarding study inclusion were resolved through discussion and consensus; when consensus could not be reached, a third reviewer (RAS) was consulted. Data extraction was performed independently by two reviewers (PAS and CC) using a predefined standardized form, and discrepancies were resolved by consensus. The PRISMA 2020 checklist can be found in the Supplementary File S1. 

### 2.2. Inclusion and Exclusion Criteria

Predefined inclusion and exclusion criteria were as follows. Eligible studies were original human research (prospective or retrospective cohorts, case–control, or diagnostic accuracy designs) that investigated rectal cancer using MRI as the primary imaging modality to evaluate both TDs and LNMs, with histopathologic confirmation following surgical resection. Only studies that provided separate diagnostic analyses or metrics for TDs versus LNMs were included. Exclusion criteria comprised studies using imaging modalities other than MRI, studies that did not explicitly distinguish TDs from LNMs, and non-primary literature (reviews, editorials, letters). Case reports and studies lacking histopathologic confirmation of lesions were also excluded, as were abstracts, dissertations, and unpublished data from registries or trial databases. The main eligibility criteria were as follows:

Inclusion:

- Original, peer-reviewed human studies
- MRI-based evaluation of rectal cancer with explicit TD vs. LNM analysis
- Surgical histopathology as the reference standard

Exclusion:

- Non-MRI imaging studies
- Studies without separate analysis of TDs and LNMs
- Mixed tumor locations or unspecified rectal involvement
- Absence of histopathologic verification
- Case reports, reviews, conference abstracts, dissertations, or registry data
- Non-English language.

All references were imported into a citation manager, and duplicates were removed using automated and manual deduplication.

### 2.3. Study Selection Results

The systematic search yielded 642 records (PubMed: 148; Embase: 203; Scopus: 177; Web of Science: 114). After deduplication, 466 unique citations remained for title and abstract screening. Of these, 442 were excluded for not meeting inclusion criteria (e.g., irrelevant cancer type, non-MRI imaging, or lack of TD-specific analysis). A total of 24 full-text articles were reviewed, from which 20 were excluded for the following reasons:

- Nine did not report tumor deposits as a distinct entity (e.g., grouped with LNMs or extramural venous invasion).
- Five employed non-MRI imaging modalities.
- Three lacked histopathologic confirmation of imaging findings.
- Two did not report diagnostic metrics separately for TD vs. LNM.
- One study was a duplicate cohort of a previously included article.

Ultimately, 4 studies met all inclusion criteria and were incorporated into the final synthesis. These studies [16,20,21,22], encompassing a total of 344 patients, used pelvic MRI as the primary modality, applied either lesion-level or patient-level diagnostic analysis of TDs versus LNMs, and included histopathologic confirmation. All four reported diagnostic performance metrics such as area under the curve (AUC), sensitivity, and specificity.

### 2.4. Risk of Bias Assessment

The methodological quality of each study was evaluated using the QUADAS-2 tool, designed for assessing risk of bias in diagnostic accuracy studies. The framework evaluates four domains: (1) patient selection, (2) conduct and interpretation of the index test (MRI), (3) reference standard, and (4) flow and timing. Each domain was assessed for risk of bias and applicability concerns, rated as “low,” “high,” or “unclear” risk based on predefined signaling questions. The results of the risk of bias assessment are summarized in both tabular and graphical formats in the Section 3.

### 2.5. Inter-Study Heterogeneity

To evaluate heterogeneity in diagnostic performance across studies, the I^2^ statistic was calculated. Standard errors were derived from reported 95% confidence intervals for AUC values. Given the small number of included studies and the methodological heterogeneity in imaging approaches, a fixed-effects model was applied for exploratory pooled estimation, acknowledging the inherent limitations of such an approach in this context. A threshold of I^2^ > 50% was considered indicative of substantial inter-study heterogeneity. Given the small number of eligible studies and the substantial clinical and methodological heterogeneity, any quantitative synthesis was considered exploratory. A fixed-effects model was applied solely for descriptive purposes, acknowledging that the assumption of a common underlying effect across studies is unlikely to be fully met in this context.

### 2.6. Heatmap Analysis of MRI Approaches

To qualitatively compare the diagnostic performance of various MRI-based techniques, a cross-modality analysis was performed. AUC values were extracted for each approach where available, including T2-weighted morphology, DWI, DCE, and radiomics-based models. For each included study, the highest AUC associated with each technique was recorded. A heatmap was generated to visually represent the relative diagnostic performance across modalities and studies.

## 3. Results

### 3.1. Overall Diagnostic Performance and Study-Level Characteristics

**Figure 2:**
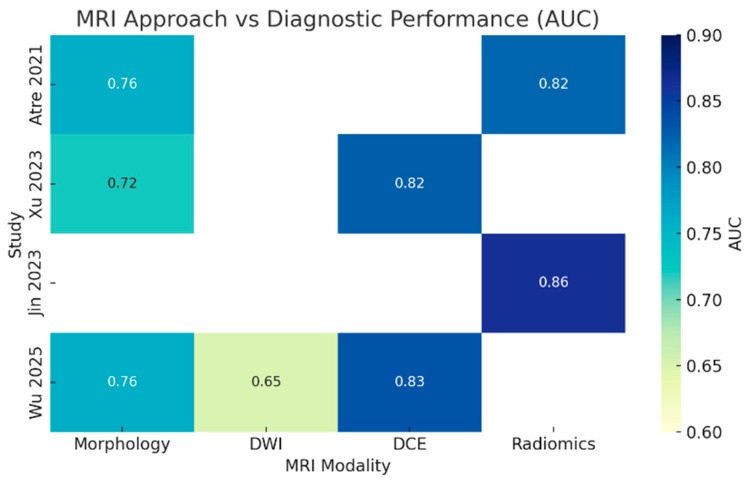
Heatmap of diagnostic performance (AUC values) for different MRI modalities across included studies. Higher AUCs indicate superior ability to differentiate tumor deposits from lymph node metastases. Empty cells indicate unavailable data for a given modality in that study. AUC, area under the curve; MRI, magnetic resonance imaging [16,20,21,22]. DWI, diffusion-weighted imaging; DCE, dynamic contrast-enhancement.

**Figure 3:**
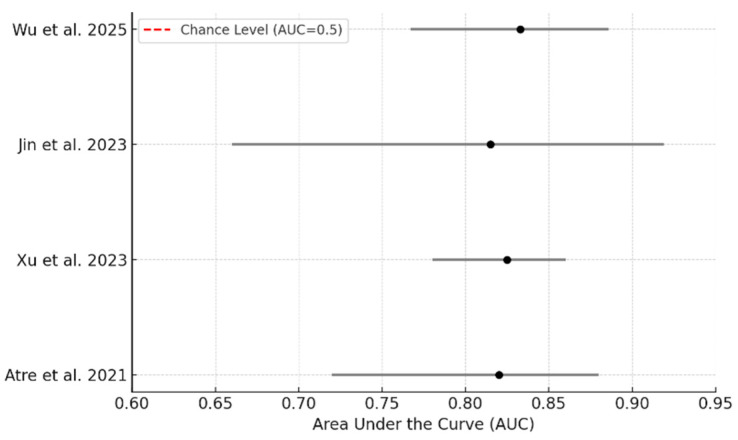
Forest plot of AUC values (using 95% confidence intervals) for the best-performing MRI model in each included study evaluating TD versus LNM differentiation [16,20,21,22].

All four included studies were retrospective, single-center investigations that correlated preoperative MRI findings with histopathologic outcomes to distinguish TDs from LNMs. Radiomics-based approaches yielded the highest diagnostic performance, with Jin et al. [16] reporting an AUC of 0.863 in the training cohort. Dynamic contrast-enhanced MRI also demonstrated robust diagnostic value, with Xu [22] and Wu [20] reporting AUCs of 0.825 and 0.833, respectively. Morphology-based models achieved moderate accuracy (AUC range: 0.76–0.82), while diffusion-weighted imaging alone showed limited discriminative capacity. A comparative heatmap (Figure 2) visually highlights the superior performance of radiomics and perfusion-based techniques across the included studies. A detailed overview of each study’s methodology and key diagnostic metrics is presented in Table 1. To address heterogeneity in study design and reporting, detailed study-level data on cohort demographics, tumor histology, MRI platforms/field strength, and the specific imaging/radiomics features used for TD–LNM discrimination are provided in Supplementary Table S1. Figure 3 displays a forest plot of the best-performing model from each study, showing a consistent range of AUC values between ~0.82 and 0.84, indicative of moderate-to-high diagnostic accuracy across MRI techniques.

**Figure 4:**
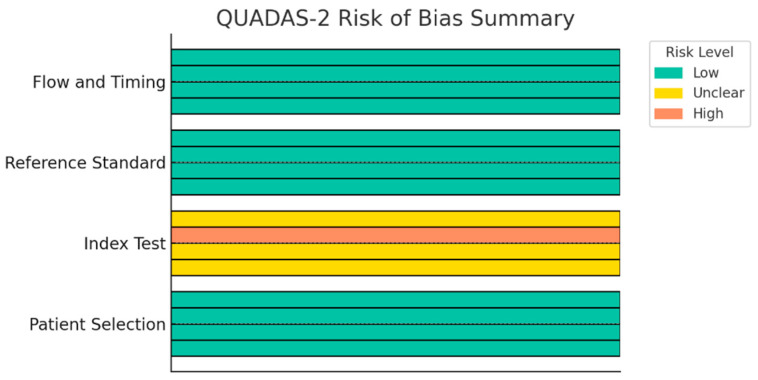
Graphical summary of QUADAS-2 risk-of-bias assessment.

Risk of bias was evaluated using the QUADAS-2 tool. Summary judgments are presented in Table 2 and Figure 4. All four studies were judged to have low risk in the patient selection domain, as they enrolled consecutive patients with histologically confirmed rectal adenocarcinoma and applied clear inclusion and exclusion criteria. Atre [21], Xu [22] and Wu [20] were rated as having unclear risk due to insufficient information on blinding procedures. Jin et al. [16] was assigned high risk, given that lesion segmentation and model training were performed retrospectively using labeled regions of interest derived from pathology, raising concerns of incorporation bias and circularity. All studies were deemed low risk in the reference standard domain. Histopathologic evaluation following surgical resection was used as the gold standard in all cases, with AJCC 8th edition criteria applied for defining TDs and LNMs. The flow and timing domain was consistently rated as low risk. All patients underwent preoperative MRI without dropouts, with a time interval of ≤4 weeks to surgery. No patients received neoadjuvant therapy during this interval that could have confounded histologic interpretation. Pooled diagnostic accuracy across the four studies yielded a fixed-effects estimate of AUC = 0.84. Inter-study heterogeneity was estimated at I^2^ = 0%; however, this finding should be interpreted cautiously, as the small number of included studies limits the power of heterogeneity statistics and precludes reliable assessment of true between-study differences.

### 3.2. MRI Characteristics by Modality

Interpretation of imaging features across studies should account for the analytical unit and case-mix. Wu et al. [20], Xu et al. [22], and Atre et al. [21] primarily performed lesion-level comparisons between TDs and LNMs/MLNs, whereas Jin et al. [16] addressed patient-level discrimination by developing a predictive model that integrates imaging-derived radiomics with clinical variables. Lesion-level designs increase statistical power but may overestimate performance if intra-patient correlation and case enrichment (e.g., inclusion of patients already known to harbor TDs and/or MLNs) are not fully addressed, whereas patient-level models face class imbalance and more closely reflect clinical decision-making.

1. Morphological Features (T2-Weighted MRI)
  - Size: Across all studies, tumor deposits demonstrated larger dimensions than lymph node metastases. Atre et al. [21] reported a significantly longer mean long axis for TDs compared to LNMs (11.7 ± 6.7 mm vs. 8.1 ± 2.0 mm). Wu and Xu [20,22] similarly found that both long and short axes were greater in TDs. Despite their larger size, TDs exhibited a lower short-to-long axis ratio, indicative of an elongated or irregular shape. Xu’s cohort [22] reported a mean ratio of 0.82 for TDs vs. 0.89 for LNMs (*p* = 0.04).
  - Margins/Borders: All studies [20,21,22] that observed the morphological features of the two lesions consistently observed that TDs exhibited more irregular or ill-defined borders compared to LNMs, which often preserved a peripheral capsule and well-defined margins [20]. Lv et al. [1] further corroborated this finding, showing that irregular margin was an independent predictor of TDs (odds ratio ~5.7). However, MRI missed ~26% of pathologically confirmed TDs, and 20% of MRI-suspected TDs were false positives.
  - Shape: Irregular shape was reported in all three studies [20,21,22] as being more characteristic of TDs, with Atre et al. noting that none of the TDs in their cohort appeared round. Irregular shape emerged as the most sensitive individual morphological predictor of TDs (sensitivity 90%), although with moderate specificity (68%). Nonetheless, a notable proportion of TDs were described as oval in shape. In contrast, LNMs were more frequently round or elliptical in all studies.
  - T2 Signal Homogeneity: Findings regarding the lesions’ intrinsic appearance were inconsistent across studies. Atre et al. [21] found that heterogeneous signal was more common in LNMs (44 lesions) than TDs (18 lesions) (*p* = 0.015). Wu [20], however, observed greater heterogeneity in TDs (42 TDs vs. 36 LNMs), while Xu [22] reported no statistically significant difference (*p* = 0.057).
  - Comet-Tail Sign: Initially proposed as an imaging feature of TDs—particularly those originating via extramural venous invasion (EMVI) [23]—the comet-tail sign was observed infrequently. Rutegård et al. found it in only 4.8% of mesorectal nodules near the primary tumor, with limited diagnostic specificity: 45.5% were benign LNMs, 36.4% TDs, and 13.6% malignant LNMs.

Collectively, conventional T2-weighted MRI features suggest that TDs are more likely when a nodule is relatively large, irregular or elongated, with indistinct margins and heterogeneous internal signal. LNMs are generally smaller, well-defined, and round or elliptical. Nonetheless, the overall discriminatory power of morphological features is modest (AUC range: 0.70–0.76), with irregular shape and spiculated margins offering the greatest specificity. Regarding morphological features’ reproducibility, this aspect was only analyzed by Wu et al. [20] who observed a substantial interreader agreement for shape (κ = 0.69) and near-perfect one for margin definition and signal heterogeneity (κ = 0.84–0.94). However, reproducibility of the short-to-long axis ratio was poor (intraclass correlation coefficient = 0.24), highlighting limited reliability of size-based criteria. A clinically relevant nuance is that the strongest single morphologic discriminator across studies—irregular or non-round configuration—likely reflects the “non-nodal” growth pattern of TDs (infiltrative tumor foci within mesorectal fat) rather than simple size enlargement. However, reliance on morphology alone remains vulnerable to overlap with irregular metastatic nodes and to reader subjectivity. This limitation is consistent with the rationale of all three “best models” in the included literature, which showed improved performance when adding quantitative surrogates (perfusion- or diffusion-related parameters in Xu and Wu [20,22], or texture/radiomics features in Atre and Jin [16,21]) to reduce dependence on subjective visual categorization.

2. Diffusion-Weighted Imaging

Diffusion-weighted imaging provides information on tissue cellularity through apparent diffusion coefficient (ADC) measurements. Tumor tissues with high cellular density generally exhibit restricted diffusion and low ADC values [24]. Two studies [20,22] evaluated whether ADC values could distinguish TDs from LNMs. Both found no statistically significant differences in absolute ADCmin, ADCmean, or ADCmax between the two lesion types. This result was likely due to the shared malignant nature of TDs and LNMs, which are both characterized by dense cellularity and reduced extracellular space [20,25]. Notably, Xu [22] excluded nodules < 5 mm, reducing variability related to small LNs with unreliable ADC measurements. Xu et al. [22] further proposed a normalization approach by comparing lesion ADC values to those of the primary tumor. They calculated lesion-to-tumor ADC ratios, revealing that TDs had lower normalized ADCmin and ADCmean ratios compared to LNMs (mean ADCmin ratio: 0.81 vs. 0.93; *p* < 0.01). This supports the biological rationale that TDs may mirror the diffusion properties of the primary tumor more closely than LNMs do.

3. Contrast-enhanced MRI

Conventional post-contrast MRI sequences had no ability to differentiate between the two entities. In one study [21], no statistically significant difference in contrast enhancement was observed (*p* = 0.37), although LNMs were nearly three times more likely to show appreciable enhancement than TDs. This discrepancy may reflect the preserved vascular supply within nodal tissue, which facilitates contrast uptake, whereas TDs often lack organized vascular architecture.

Dynamic contrast-enhanced MRI, by capturing temporal patterns of gadolinium distribution, offers a more refined assessment of tissue perfusion and permeability [26]. The perfusion profiles of TDs and LNMs are expected to differ due to their divergent histopathological origins, with TDs arising from perivascular tumor spread and lacking structured nodal vessels, whereas LNMs reside within pre-existing lymphatic structures. This theoretical distinction has been supported by quantitative analyses in two studies [20,22]. Xu et al. [22] extracted semi-quantitative enhancement parameters, including relative enhancement (RE) and maximum relative enhancement (MRE), for each lesion. Both metrics were significantly lower in TDs than in LNMs, consistent with lower vascular perfusion. RE reflects the cumulative gadolinium diffusion into both intravascular and interstitial compartments, while MRE corresponds to the peak enhancement at the time of maximal tissue uptake [26]. Interestingly, prior studies have shown that RE and MRE correlate with hypoxia-inducible factor-1α expression, a surrogate marker of tumor angiogenesis [27]. However, in absolute terms, RE and MRE alone showed only modest diagnostic power: sensitivity and specificity were 73.3% and 55.2% for RE, and 86.7% and 55.2% for MRE, respectively. Xu et al. [22] subsequently introduced normalized ratios (comparing lesion values to those of the primary tumor) and demonstrated that diagnostic accuracy improved substantially. The two DCE-based studies also highlight that “DCE-MRI” is not a uniform technique: Xu et al. [22] relied on semi-quantitative enhancement metrics and lesion-to-tumor ratios (e.g., RE-ratio, MRE-ratio), whereas Wu et al. [20] implemented quantitative pharmacokinetic modeling and identified v_e_ as the strongest single discriminator. These findings are biologically coherent—TDs may exhibit less organized microvascular architecture and more stromal/extracellular components—yet the differing DCE implementations imply that reported thresholds (e.g., v_e_ cut-offs or enhancement ratios) are unlikely to be directly transferable across institutions without protocol harmonization and external validation. The RE-ratio achieved an AUC of 0.749, with sensitivity of 83.3% and specificity of 72.4%. The MRE-ratio also contributed diagnostically, reaching 70% sensitivity and 75.9% specificity. When both parameters were combined in a multivariate model, the overall AUC increased further to approximately 0.74. These findings suggest that TDs are enhanced less vividly and more heterogeneously than LNMs, likely due to the absence of structured microvascular beds and the presence of tumor fibrosis.

**Figure 5:**
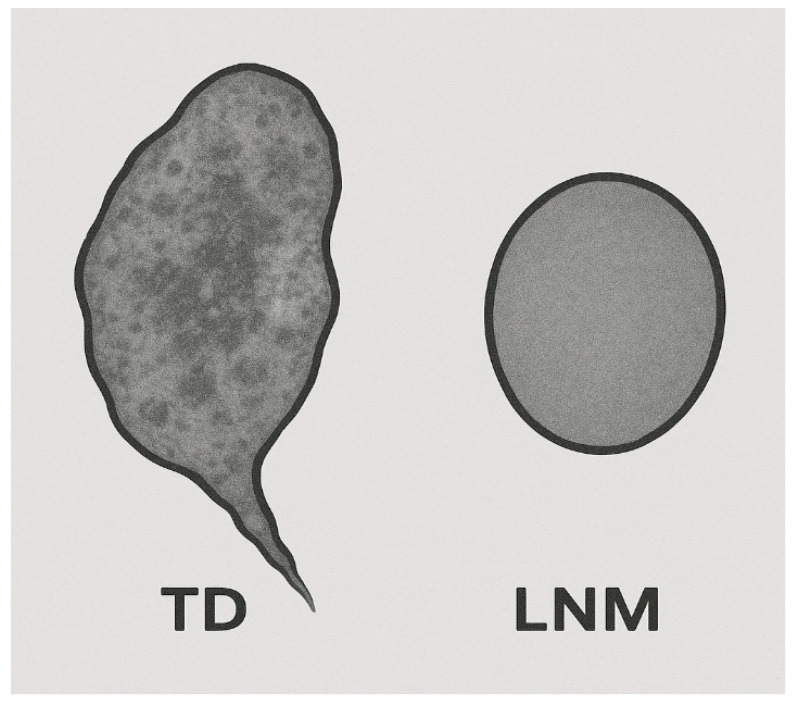
Schematic contrast between tumor deposit (TD) and lymph node metastasis (LNM) in rectal cancer. TDs (**left**) are depicted with irregular, spiculated contours and heterogeneous internal signal intensity, often consistent with stromal infiltration and vascular invasion. LNMs (**right**) appear as rounded, well-defined structures with homogeneous signal and smooth borders. This figure was generated using AI-based synthesis in ChatGPT (version 5.1, OpenAI, San Francisco, CA, USA) for illustrative academic use only, and represents a conceptual illustration based on published morphological descriptors and does not derive from clinical data.

Complementary pharmacokinetic modeling was explored by Wu et al. [20], who analyzed classic parameters such as the volume transfer constant (K_trans_), rate constant (K_ep_), and fractional extravascular extracellular space (v_e_). Their results revealed that TDs were characterized by higher v_e_ and K_tran_ values, but lower K_ep_ values, when compared to LNMs. The elevated v_e_ could reflect desmoplastic stroma or increased interstitial space associated with invasive growth patterns. Elevated K_trans_ may be a marker of increased angiogenesis, while the reduced K^ep^ might correspond to impaired vascular return or diminished microvascular density. Of these, v_e_ demonstrated the best individual discriminative capacity, achieving an AUC of 0.772 for differentiating TDs from LNMs. A threshold of v_e_ 0.246 yielded sensitivity and specificity of 73% and 72%, respectively. In contrast, K_tran_ alone had weaker performance, showing high sensitivity (87%) but low specificity (32%), suggesting substantial overlap between TDs and LNMs in terms of permeability alone. Key MRI features distinguishing TDs from nodes are summarized in Table 3. The schematic illustration in Figure 5 complements this summary by visually contrasting prototypical appearances of TDs and LNMs on high-resolution MRI.

4. Multiparametric MRI Models and Radiomic Analysis

Wu et al. [20] proposed a multiparametric MRI model combining morphological, DWI and DCE features. Among the evaluated parameters, short-axis diameter, border definition, and v_e_ were independently associated with the presence of tumor deposits. The combined model achieved a sensitivity of 72.9%, specificity of 83.7%, and an AUC of 0.833, demonstrating significantly improved diagnostic performance over individual features. Xu et al. [22] adopted a similar multiparametric strategy, incorporating four normalized imaging parameters derived from both ADC and DCE sequences: ADCmin-ratio, ADCmean-ratio, RE-ratio, and MRE-ratio. This model achieved an AUC of 0.825, exceeding the performance of any single modality. Although the study did not report discrete sensitivity and specificity values for the composite model, the overall accuracy approached 82%, and graphical interpretation of the ROC curve suggested a balanced trade-off between sensitivity and specificity in the range of 80%. Taken together, the multiparametric models in Wu and Xu [20,22] suggest that clinically useful TD–LNM discrimination may require combining orthogonal information sources: morphology (shape/border/size), perfusion-related behavior and diffusion-related behavior. The fact that the best single parameter differed between these studies further supports the interpretation that performance is sensitive to protocol design and parameterization. Practically, this argues for reporting strategies that prioritize robust qualitative descriptors (e.g., irregularity and ill-defined margins) and reserve quantitative thresholds for settings where acquisition is standardized.

Two studies [16,21] investigated the added value of radiomics in differentiating TDs from LNMs, using texture features extracted from T2-weighted images. Atre et al. [21] focused on first-order radiomic features and identified skewness (measured on fine-scale filtered T2-weighted image) as a significant discriminator. Skewness, a measure of histogram asymmetry, was significantly lower in TDs (median 0.11) compared to LNMs (median 0.40), with corresponding sensitivity and specificity of 70% and 72%, respectively. Although the authors did not provide a radiological interpretation of this result, skewness likely reflects the overall brightness and homogeneity of lesion signal. Thus, TDs may exhibit lower signal intensity or greater intralesional darkness on T2-weighted sequences, consistent with prior findings that TDs show lower T2 signal than the primary tumor [1]. Notably, when skewness was combined with lesion shape, the composite model demonstrated a substantial increase in discriminative ability, achieving 91% sensitivity and 68% specificity, with a combined AUC of 0.82—markedly higher than that of morphological analysis alone (AUC 0.76). These results support the broader principle that hybrid models combining multiple domains of image-derived information outperform single-parameter analysis. An additional interpretive point is that the incremental gain from texture/radiomics in Atre et al. [21] was achieved using a single, biologically plausible first-order descriptor (skewness) combined with lesion shape, suggesting that even low-dimensional quantitative features can meaningfully complement morphology. This contrasts with high-dimensional radiomics pipelines, where performance gains may reflect both true signal and increased risk of overfitting, especially in single-center retrospective cohorts.

The study by Jin et al. [16] represented the most comprehensive radiomics-based approach among the four, employing high-dimensional texture extraction from T2-weighted images across seven radiomic classes. The authors developed a robust radiomics score (Rad-score) capable of discriminating between TDs and LNMs with 69.5% sensitivity and 79.8% specificity in the training cohort, and 86.7% sensitivity with 69.2% specificity in the validation cohort. Diagnostic performance improved further when the radiomic model was combined with clinical variables, including carbohydrate antigen 19-9 (CA 19-9) levels, MRI-reported nodal stage, and tumor volume, achieving 94.9% sensitivity and 62.5% specificity in the training cohort, and 93.3% sensitivity and 73.1% specificity in the validation set. Overall, the highest AUCs across all studies (range 0.82–0.86) were attained by such integrated multiparametric models, with sensitivities ranging from 70 to 95% and specificities between 62 and 84%, depending on threshold definitions and cohort composition. Among the studies included in this review, the model proposed by Jin et al. [16] demonstrated the highest overall diagnostic accuracy for differentiating tumor deposits from lymph node metastases, with an AUC of 0.863, sensitivity of 84.8%, and specificity of 75.8% when combining radiomic and clinical data. These results support the integration of radiomics into personalized pre-treatment decision-making frameworks, where combined radiomic-clinical scores may function as non-invasive surrogates for histopathologic risk markers. However, interpretation of the apparent superiority of the combined nomogram should be tempered by study design: the model was internally validated but not externally tested, and the radiomics pipeline relied on extensive preprocessing and feature extraction from both tumor and peritumoral fat on T2-weighted images [16]. The inclusion of peritumoral fat features is clinically intriguing, as it may capture microinvasion or stromal reaction beyond the visible nodule, but it also increases dependence on consistent acquisition and segmentation. Therefore, the clinical value of such models is best viewed as hypothesis-generating until validated across independent scanners, institutions, and patient populations.

A related study by Yan et al. aimed to develop a preoperative risk prediction model by integrating radiomic features extracted from the primary rectal tumor with conventional clinical and imaging data. While the resulting nomogram achieved good predictive performance, it did not directly assess mesorectal nodules themselves, instead extrapolating nodal and TD status based solely on the characteristics of the primary tumor. Similar modeling strategies have been reported by other groups [28,29,30,31], with varying degrees of validation and generalizability. However, these studies fall outside the scope of the present review due to the lack of lesion-specific imaging-pathology correlation.

## 4. Discussion

This systematic review consolidates evidence on the diagnostic differentiation of TDs and LNMs in rectal cancer using MRI. While conventional sequences yield overlapping features, advanced imaging approaches increasingly facilitate more confident discrimination. Beyond imaging, however, the biological significance and prognostic role of TDs remains a critical area of debate. Importantly, the four included studies achieved broadly comparable discriminative performance (AUC approximately 0.82–0.86 for the best-performing models), despite using markedly different strategies—morphologic assessment alone, multiparametric functional MRI, and radiomics-based modeling [16,20,21,22]. This convergence suggests that TD–LNM separation is not driven by a single “optimal” sequence, but by partially overlapping imaging signatures of tumor biology that can be captured through different methodological lenses. Consequently, differences in study design and protocol implementation become critical when interpreting apparent performance advantages between approaches.

The superior diagnostic performance of multiparametric models integrating DWI, DCE-MRI, and radiomics can be explained by the complementary biological information captured by each technique. Although tumor deposits and metastatic lymph nodes are both malignant perirectal nodules, prior pathological and imaging studies have demonstrated fundamental differences in their pathogenesis, growth pattern, and interaction with the surrounding mesorectal tissue, which are reflected in their imaging behavior.

Diffusion-weighted imaging primarily reflects tissue cellularity, which shows substantial overlap between tumor deposits and metastatic lymph nodes, thereby limiting the discriminatory value of absolute ADC metrics when used in isolation. This overlap has been consistently reported, including in studies where no significant differences in absolute ADC values were observed between the two entities [19,21]. In contrast, relative diffusion metrics, particularly lesion-to-primary-tumor ratios, appear more informative, as tumor deposits tend to share diffusion characteristics with the primary tumor rather than with nodal metastases [21].

Dynamic contrast-enhanced MRI provides additional insight into microvascular permeability and the extravascular extracellular space. Quantitative DCE-MRI analysis has identified parameters such as the extracellular volume fraction (v_e_) as key discriminators between tumor deposits and metastatic lymph nodes [19], likely reflecting infiltrative stromal growth and altered microvascular properties in tumor deposits compared with the more organized vascular architecture of lymph nodes. Similar differences in enhancement dynamics have been reported in semi-quantitative DCE-MRI analyses [21].

Radiomics further complements functional imaging by quantifying intralesional heterogeneity and spatial irregularity. Texture-based analysis on T2-weighted imaging has shown that features reflecting asymmetry and heterogeneity are associated with tumor deposits, consistent with their infiltrative growth pattern [16]. More complex radiomics-based models integrating tumor and peritumoral features have demonstrated additional gains in diagnostic performance, particularly when combined with conventional MRI and clinical variables [15].

Collectively, these findings indicate that multiparametric approaches outperform single-sequence models by integrating non-redundant imaging surrogates of tumor biology, encompassing cellularity, perfusion characteristics, extracellular composition, and spatial heterogeneity. This integrative framework provides a biologically grounded explanation for the improved discrimination between tumor deposits and metastatic lymph nodes consistently observed across studies [15,16,19,21].

### 4.1. Tumor Deposits: Implications for Staging, Prognosis, and Clinical Management

Multiple theories have been proposed regarding the origin of TDs, including vascular dissemination [32], lymphatic spread with complete replacement of nodal architecture [14], perineural invasion, and discontinuous extension of the primary tumor into mesorectal fat [23,33]. The anatomical contiguity of vascular, lymphatic, and neural channels likely explains why TDs often contain components from multiple structures, particularly in larger lesions [34]. The 8th edition of the TNM classification (TNM8, 2017) defines “pure” TDs as isolated nodules of adenocarcinoma in the pericolorectal fat, located within the lymphatic drainage area of the primary tumor, and lacking histological evidence of residual lymphoid, vascular, or neural tissue [10]. In cases where a vascular wall or neural remnant is identified histologically, the nodule is reclassified as venous, lymphatic, or perineural invasion (which are termed “non-nodular” or “invasive” TDs) [4]. According to TNM8, “pure” TDs do not influence the T category but change nodal status to pN1c if all regional LNs are negative. However, for patients with concurrent TDs and LNMs, the impact of TDs on staging or prognosis remains undefined [35]. By contrast, the Japanese Society for Cancer of the Colon and Rectum (JSCCR) guidelines (2013, 2018) treat “pure” TDs as metastatic lymph nodes, counting each deposit individually toward the total nodal count. Invasive TDs (those with identified vessel or nerve remnants) contribute instead to the T stage [36], thereby altering both pT and pN classification and potentially enhancing staging granularity.

Nevertheless, the histological subclassification of TDs has been challenged. Ueno et al. [4] argue that most prognostic studies on TDs did not exclude deposits with remnant structures, and there is no compelling evidence that such exclusions improve staging accuracy. From a biological standpoint, all isolated mesorectal nodules—whether ultimately classified as LNs or TDs—are thought to result from tumor migration through vascular, lymphatic, or perineural pathways, regardless of whether these channels remain visible microscopically. Thus, assigning clinical meaning based solely on assumed histogenesis may introduce unnecessary complexity and obscure prognostically relevant features.

### 4.2. Pathological and MRI Controversies

1. Tumor Deposits vs. Lymph Node Metastases

The histopathologic distinction between TDs and LNMs remains problematic and often lacks reproducibility, even among experienced gastrointestinal pathologists. A multicenter study conducted by the Japanese Society for Cancer of the Colon and Rectum (JSCCR) across 11 hospitals reported a moderate interobserver agreement for differentiating TDs from LNMs, with a κ value of 0.74 [37]. In a separate investigation, Rock et al. found that consensus among expert pathologists using AJCC 7th edition criteria was achieved in fewer than 50% of cases [38]. These findings emphasize the diagnostic ambiguity and subjective nature of histological criteria currently employed to distinguish between these entities.

2. Tumor Deposits and Extramural Vascular Invasion

An additional area of uncertainty concerns the differentiation between TDs and nodular forms of extramural vascular invasion, both of which have significant prognostic implications. EMVI is defined histologically as tumor infiltration of veins beyond the muscularis propria [39]. However, vascular wall destruction by tumor desmoplasia often obliterates identifiable venous anatomy, complicating pathological assessment [39]. Some investigators argue that certain TDs represent fully evolved EMVI—i.e., intravascular tumor extensions in which the vessel wall has been destroyed and is no longer discernible histologically [40,41]. This concept aligns with theories suggesting that TDs are extravascular manifestations of EMVI, forming as perivascular deposits following transmural vascular infiltration [42,43,44]. Other authors adopt a more cautious view, acknowledging that although EMVI increases the likelihood of TD development, the two remain pathologically distinct [45]. Moreover, prognostically, only TDs—rather than EMVI—have consistently demonstrated independent predictive value for disease-free survival [46], suggesting a more adverse biological behavior.

MRI offers unique advantages in evaluating vascular involvement. EMVI is classically visualized as irregular tumor signal extending into mesorectal vessels on T2-weighted sequences [34,47]. In parallel, recent radiological lexicons [14] and MRI-based studies [10,23] have characterized TDs as nodular lesions with tapering or continuity with vessels on orthogonal planes, irregular margins, and comet-tail morphology—features typically attributed to vascular-derived TDs. However, this raises a critical question: how should radiologists identify TDs that lack vascular contact, such as those arising from perineural spread or discontinuous invasion? To address these challenges, Huang et al. [34] proposed a unified MRI scoring framework that integrates TDs and EMVI into a single radiologic construct (mrTD/EMVI), reflecting the overlapping morphology and potential biological continuum between these two processes. Their justification rests on the hypothesis that TDs and EMVI may represent different manifestations of the same invasive pathway. Nonetheless, they acknowledge prognostic distinctions: in their study, 20.8% of TD-positive patients lacked EMVI, supporting the possibility of alternative mechanisms of TD formation [23,48].

### 4.3. Challenges and Future Research Directions

Despite promising advances, several key challenges remain in refining the radiologic identification of tumor deposits (TDs) in rectal cancer.

1. Standardization of Imaging Criteria. Across the reviewed literature, there is considerable variability in the imaging criteria used to define TDs. This heterogeneity hampers comparability and limits clinical translation. Prospective, multicenter validation studies are needed to establish reproducible and clinically meaningful criteria. The ongoing COMET trial is a critical step in this direction, aiming to correlate MRI-detected mesorectal nodules with surgical pathology and define standardized imaging features for TDs versus lymph node metastases. Establishing a standardized MRI reporting approach for TD-suspicious nodules will require professional consensus, structured lexicons, and dedicated educational efforts within the radiology community.

2. External Validation and Generalizability. Most available studies are retrospective and single-center in design, inherently limiting their external validity. Across the included studies, MRI acquisition heterogeneity was substantial and extends beyond sequence selection to differences in scanner platforms, field strength, and parameterization, with direct implications for reproducibility. Although Wu et al. [20] and Xu et al. [22] employed dedicated multiparametric rectal MRI protocols (high-resolution T2-weighted imaging combined with DWI and DCE), they used clearly different acquisition strategies for DWI (b-value schemes and derived ADC handling) and for DCE (implementation and temporal sampling/parameter derivation), which can influence the stability of quantitative readouts. Importantly, both Atre et al. [21] and Jin et al. [16] analyzed data acquired on more than one scanner (including multi-vendor 3T imaging), thereby introducing additional variance related to hardware, coils, reconstruction, and voxel geometry [16,21]. This is particularly relevant for texture/radiomics pipelines, as feature values are sensitive to scanner- and protocol-dependent factors (e.g., spatial resolution, SNR, intensity scaling), and such variability may inflate within-center performance while reducing external generalizability. Collectively, these acquisition differences represent a plausible source of limited method transferability across institutions and reinforce the need for standardized acquisition frameworks and/or rigorous harmonization with external validation before TD–LNM discrimination approaches can be translated into routine practice [16,20,21,22]. This highlights the urgent need for multicentric data-sharing initiatives and collaborative model training to ensure robustness across platforms and demographics.

3. Radiology–Pathology Correlation. Effective cross-disciplinary communication is essential for improving diagnostic consistency. Pathologists may miss small tumor deposits unless specifically targeted, while radiologists may detect suspicious nodules not documented in pathology reports. Lord et al. [15] addressed this issue in a high-quality correlation study of 609 mesorectal nodules, revealing that 44% of nodal-appearing structures on MRI were, in fact, tumor deposits or extranodal tumor tissue on histopathologic analysis. Similar integrative studies are needed to calibrate radiologic interpretation against true histologic outcomes.

4. Clinical Translation of Radiomics and artificial intelligence. Although radiomics-based models have shown encouraging results, their integration into clinical workflows remains limited. Challenges include the need for harmonized acquisition protocols, standardized lesion segmentation, and interpretability of radiomic features. Additionally, variations in MRI quality or protocol parameters can introduce substantial noise into feature extraction, potentially undermining diagnostic reliability. Before these tools can be adopted in routine practice, they must undergo technical validation and clinical trial-based evaluation. Moreover, most published radiomics and artificial intelligence models in this setting are derived from small, single-center cohorts, lack external validation, and are therefore susceptible to overfitting, which currently limits their robustness and precludes direct clinical translation.

5. Pathology-Informed Imaging Phenotyping. A novel direction for future research involves investigating whether distinct histopathological subtypes of TDs (e.g., vascular, neural, or fibrotic) correspond to specific and reproducible MRI phenotypes. Such correlations could enhance the specificity of radiologic interpretation and refine prognostic stratification. In this context, we propose an exploratory framework, termed the Imaging Complexity Score (ICS), built to stratify mesorectal nodules based on morphological traits frequently associated with tumor deposits. The ICS ranges from 0 to 3, assigning one point each for the presence of (1) irregular or spiculated contour, (2) ill-defined margins, and (3) adjacency to mesorectal vessels. This score is not intended as a diagnostic classifier but rather as a standardized descriptor to facilitate research hypotheses and improve interobserver reporting consistency. Future studies are needed to validate its prognostic and diagnostic utility through histopathology-confirmed imaging-pathology correlation.

6. Personalized diagnosis and treatment. Personalized medicine relies on the capacity to characterize disease heterogeneity in a clinically actionable manner. The reviewed radiomic and functional MRI parameters represent potential non-invasive biomarkers for stratifying mesorectal disease aggressiveness. Incorporating these metrics into predictive models may allow selection of patients who are likely to benefit from intensified treatment versus those eligible for de-escalation. A key priority will be to integrate imaging phenotypes with genomic and pathological data in future multimodal frameworks.

### 4.4. Limitations

This review is subject to several limitations that must be acknowledged when interpreting its findings. First, all included studies were retrospective and conducted at single institutions, with sample sizes ranging from 30 to 204 patients. These characteristics limit generalizability and introduce potential selection bias. The selective inclusion of patients with pathologically confirmed tumor deposits may have resulted in a spectrum bias, favoring larger or morphologically distinct lesions and underrepresenting smaller, ambiguous nodules that are more difficult to identify on MRI. Second, considerable heterogeneity was observed in MRI acquisition protocols across studies, including differences in field strength (1.5 Tesla vs. 3 Tesla), coil configuration, spatial resolution, and sequence parameters. Although intra-study comparisons (i.e., TDs vs. LNMs within the same MRI protocol) minimize technical confounding at the individual study level, these variations hinder inter-study comparability and preclude reliable meta-analytic synthesis of diagnostic performance. Third, there were minor but notable discrepancies in the histopathological definitions of tumor deposits. While most studies adhered to AJCC 8th edition criteria, others, such as Wu et al., included mesorectal nodules exceeding a certain size without overt nodal characteristics, potentially broadening the inclusion criteria. Moreover, the reproducibility of histologic distinction between TDs and LNMs remains suboptimal, with reported interobserver agreement in the literature being only moderate. This variability affects the consistency of the reference standard across studies. Fourth, statistical handling of lesion-level data varied significantly. While some investigators, such as Atre [21] and Wu [20], accounted for within-subject lesion clustering, others like Xu [22] and Jin [16] treated each lesion as an independent event, potentially inflating diagnostic metrics due to violation of statistical independence. Additionally, many studies failed to stratify results by lesion size or type, despite potential relevance for MRI detectability and classification accuracy. Fifth, the majority of included patients underwent MRI prior to any neoadjuvant chemoradiotherapy, which limits the applicability of findings to modern clinical workflows. As preoperative treatment is now frequently administered in rectal cancer, the impact of therapy on the appearance and detectability of TDs on MRI remains insufficiently addressed and represents an important area for future research. Sixth, although radiomics and machine learning techniques showed promising results, their clinical integration remains premature. Current models are susceptible to overfitting, lack external validation, and depend on reproducible segmentation and acquisition protocols. The generalizability of such tools across scanners, institutions, and populations remains to be demonstrated. Finally, this review did not conduct a quantitative meta-analysis due to substantial clinical and methodological heterogeneity across studies, including differences in MRI protocols, diagnostic criteria, and analytical approaches. Although heterogeneity statistics such as the I^2^ metric were explored, their interpretability is limited in the context of a small number of included studies, where such tests are underpowered and may fail to detect true between-study variability. Accordingly, sensitivity, specificity, and AUC values were synthesized narratively rather than pooled quantitatively. While this approach limits statistical precision, the convergence of findings across independent studies supports the robustness of the observed diagnostic trends. Therefore, all quantitative findings should be interpreted as exploratory rather than confirmatory.

## 5. Conclusions

This systematic review suggests that multiparametric MRI approaches, including functional imaging and exploratory radiomics-based analyses, may offer potential for improving the preoperative differentiation between tumor deposits and nodal metastases in rectal cancer. However, the current evidence remains limited by the small number of available studies and methodological heterogeneity. Future prospective, multicenter studies with standardized imaging protocols and external validation are required to determine whether these imaging biomarkers can reliably contribute to personalized staging and treatment decision-making in rectal cancer.

## Figures and Tables

**Table 1:** Characteristics of included studies and diagnostic performance metrics for MRI-based differentiation of tumor deposits (TDs) versus lymph node metastases (LNMs) in rectal cancer.

Author, Year	Number Patients	Analysis Approach	Diagnostic Accuracy		
			AUC	Se	Sp
WU et al., 2025 [20]	70	Ve	0.772	72.9	72.4
		Combined model of morphological features, DCE and ADC	0.833	72.9	83.7
XU et al., 2023 [22]	30	RE-ratio	0.749	83.30	72.4
		Combined model of DCE and DWI	0.825	83–85 *	75–77 *
D.Atre et al., 2021 [21]	40	Lesion shape	0.76	90	68
		Skewness	0.7	70	72
		Combined lesion shape and skewness	0.82	91	68
Jin et al., 2023 [16]	204	Radiomic score—training cohort	0.812	69.5	79.8
		Radiomic score—validation cohort	0.792	86.7	69.2
		Combined model—training cohort	0.863	94.9	62.5
		Combined model—validation cohort	0.815	93.3	73.1

* Estimates inferred from receiver operating characteristics graphics due to incomplete numerical reporting. AUC, area under the curve; Se, sensitivity; Sp, specificity; Ve, the fractional extravascular extracellular space volume; RE, relative enhancement; MRE, maximal relative enhancement; DWI, diffusion-weighted imaging; DCE, dynamic contrast-enhancement.

**Table 2:** QUADAS-2 assessment summary.

Domain	Atre 2021 [21]	Xu 2023 [22]	Jin 2023 [16]	Wu 2025 [20]
Patient Selection	Low Risk	Low Risk	Low Risk	Low Risk
Index Test	Unclear Risk ^1^	Unclear Risk ^1^	High Risk ^2^	Unclear Risk ^1^
Reference Standard	Low Risk	Low Risk	Low Risk	Low Risk
Flow and Timing	Low Risk	Low Risk	Low Risk	Low Risk

^1^ Blinding to histopathology not reported. ^2^ Radiologists likely aware of lesion status due to retrospective training on annotated regions of interest.

**Table 3:** Summary of MRI Features for Differentiating Tumor Deposits from Lymph Node Metastases, based on the results of the selected studies [16,20,21,22].

MRI Feature	Tumor Deposit	Lymph Node Metastasis
Shape	Irregular, spiculated	Round or oval (smooth)
Margin	Ill-defined borders	Well-defined borders
Short-axis diameter	Larger; lower short/long axis ratio	Smaller; higher ratio
Signal heterogeneity (T2)	More heterogeneous (e.g., higher skewness)	More homogeneous
Contrast enhancement (DCE)	Lower RE and MRE	Higher peak enhancement
Diffusion (ADC)	Lower minimum and mean ADC values	Higher ADC (less restricted diffusion)
Location (vascular adj.)	Often adjacent to mesorectal vessels (high EMVI)	Less frequently adjacent to vascular bundles

## Data Availability

Data sharing is not applicable, since the article is a review of the literature.

## Supplementary Materials

The following supporting information can be downloaded at: https://www.mdpi.com/article/10.3390/jcm15041390/s1, Supplementary File S1: PRISMA 2020 Checklist [49]. Table S1: Study-level cohort characteristics, tumor histology, MRI acquisition platforms, and imaging/radiomics features used for preoperative differentiation of tumor deposits and lymph node metastases
