# Supplementary material for: MRI-Based Differentiation of Tumor Deposits and Lymph Node Metastases in Rectal Cancer: A Systematic Review of Diagnostic Performance
Source: J Clin Med. 2026 Feb 10;15(4):1390. doi: 10.3390/jcm15041390 (PMC12941611; doi:10.3390/jcm15041390)
Supplement: Supplementary file 1 [file jcm-15-01390-s001.zip › table s1.pdf]

Supplementary Table S1. Study-level cohort characteristics, tumor histology, MRI acquisition platforms, and imaging/radiomics features used for preoperative differentiation of tumor deposits and lymph node metastases.

Author, year	Patient Demographics (Age, Sex)	Tumor histology	MRI field strenght and parameters	Paramters used for differentiating deposits and lymph node metastases
WU et al, 2024 [20]	Mean 58.6 ± 12.6 years; 49% male.	Adenocarcinoma (excluded othe histologies)	3T scanner (Magnetom Verio; Siemens Healthcare, Erlangen, Germany)	*Morphological features*: size, shape, border, T2 signal intesity.<br>*DCE parameters*: Ktrans, kep, and ve<br>*DWI parameters*: ADCmin, ADCmax, and ADCmean
XU et al, 2023 [22]	Mean 60.5 years (33–74 years); 53.33% % male.	Adenocarcinoma (excluded othe histologies)	3T scanner (Ingenia, Philips Medical Systems)	Morphological features: size, shape, border, internal heterogenity.<br>*DCE parameters*: relative enhancement, maximum enhancement, maximum relative enhancement, time to peak, wash-in rates, wash-out rates, brevity of enhancement.<br>*DWI parameters*: ADCmin, ADCmax, and ADCmean
D.Atre et al, 2021 [21]	Median: 56.1 years (34–82 years); 47 % male.	Not mentioned	3T (General Electric Discovery) and 1.5 T (Siemens – exact model was not specified)	*Morphological features*: lesion shape, size, signal heterogeneity, contrast enhancement.<br>*First-order texture features* (mean, standard deviation, skewness, entropy, kurtosis, mean value of positive pixels)
Jin et al., 2023 [16]	Mean 58.28 ± 12.39 years; 63.1% male.	Adenocarcinoma (excluded othe histologies)	3T (MAGENTOM Skyra, Siemens Healthcare Sector, Germany.<br>SIGNA Architect, GE Medical System, America; Ingenia Elition X, Philips, Netherlands; UMR790, UIH, China	First-order texture features (gray-level size zone matrix; gray-level co-occurrence matrix; gray-level dependence matrix; gray-level run-length matrix; and neighboring gray tone difference matrix)

T, Tesla.
